# Novel Heterozygous Mutations in* JAG1* and* NOTCH2* Genes in a Neonatal Patient with Alagille Syndrome

**DOI:** 10.1155/2017/1368189

**Published:** 2017-03-29

**Authors:** Alisa Brennan, Anil Kesavan

**Affiliations:** ^1^Rush Medical College, 600 S. Paulina St., Chicago, IL 60612, USA; ^2^Section of Pediatric Gastroenterology, Rush University Medical Center, Professional Building, 1725 W. Harrison Street, Suite 710, Chicago, IL 60612, USA

## Abstract

Alagille Syndrome (ALGS) is a rare autosomal dominant disorder that affects multiple organ systems. Cholestasis as a result of a paucity of intrahepatic bile ducts and congenital heart defects are the two most common features of ALGS. We describe a case of ALGS with novel mutations of* JAG1* and* NOTCH2* genes in a newborn girl with complex congenital heart disease, bilateral dysplastic kidneys, and malrotation with volvulus.

## 1. Introduction

Alagille Syndrome, also known as arteriohepatic dysplasia, is a rare autosomal dominant disorder that affects multiple organs [1, 2]. ALGS is estimated to occur in 1 in every 70,000–100,000 live births, with the prevalence underestimated due to the highly variable expressivity observed within and between families with ALGS [1, 2]. Neonatal jaundice from impaired intrahepatic bile duct formation and congenital heart disease are the two most common features of ALGS [1]. Other major and minor signs are summarized in Box 1 [1, 3, 4]. This multisystem disorder is localized to defects in the Notch signaling pathways, with the majority of cases attributed to mutations in* JAG1* gene and a smaller percentage in* NOTCH2* gene [1, 3, 5, 6].

## 2. Case Presentation

A full-term African American female with a known history of right hypoplastic heart syndrome and pulmonary hypoplasia initially presented with rising direct hyperbilirubinemia during the first 24 hours of life. Laboratory tests showed elevated total bilirubin (5.5 to 8.0 mg/dL) and direct bilirubin (2.39 to 3.87 mg/dL) with normal aspartate transaminase (81 U/L), alanine transaminase (20 U/L), alkaline phosphatase (147 U/L), PT (12.2 secs), INR (1.19), and PTT (39.3 secs). Ursodiol and AquADEKs were started. First day of life (DOL) was also complicated by apnea requiring intubation as well as gross blood in her urine with elevated creatinine and proteinuria. An abdominal ultrasound with Doppler showed bilateral dysplastic kidneys, patent renal vessels, and a solitary left renal parenchymal cyst but no hydronephrosis. The liver and biliary system appeared normal. On DOL2, the patient had increasing abdominal distention for which an upper gastrointestinal series was completed and confirmed malrotation with mid-gut volvulus. A Ladd's procedure with inversion appendectomy was subsequently performed. Initial echocardiograms were significant for a secundum ASD and a small VSD, both with right to left shunt, as well as a hypoplastic right ventricle, pulmonary valve atresia, small collateral to right pulmonary artery, and small aortopulmonary collaterals. Creatinine began to downtrend but direct bilirubin continued to increase.

Due to unresolved hyperbilirubinemia by DOL17, a HIDA scan was ordered which demonstrated normal hepatic uptake with poor flow through the biliary tree. A repeat HIDA scan revealed no flow through the biliary tree. Intraoperative cholangiogram was scheduled secondary to concern for biliary atresia. This study showed patent biliary system distal to the cystic duct with very small intrahepatic biliary ducts visualized, indicating a stenotic biliary system. Liver biopsy was significant for severe hepatocellular cholestasis with ballooning and slight deficiency of interlobular bile ducts and cholangioles suggestive of ALGS. Total and direct bilirubin continued to rise (maximum total bilirubin 32.4 mg/dL and direct 24.1 mg/dL) with no evidence of hepatic insufficiency. The patient was optimized on ursodiol with the addition of rifampin, cholestyramine, and diphenhydramine for worsening pruritus. Ophthalmic exam was normal. Dysmorphic features were not initially recognized; however, she was subsequently noted to have deep set eyes, prominent forehead, bulbous tip of the nose, hypertelorism, and prognathic chin. Genetic analysis demonstrated a pathogenic* JAG1* gene mutation as well as a* NOTCH2* gene variant of unknown significance, confirming the diagnosis of ALGS. Patient was determined not to be a surgical or transplant candidate, given her extensive cardiac disease, with no additional medical treatments available to extend her life. She was eventually transitioned to hospice care at 3 months of age and subsequently passed away at 6 months of age.

## 3. Discussion

ALGS is traditionally diagnosed using clinical guidelines that are known as the classic criteria (Box 2) [1, 3, 7]. Chronic cholestasis occurs in a high proportion of cases (~97%) and typically presents as jaundice due to conjugated hyperbilirubinemia in early infancy, like our patient [1–3, 5]. The insufficient bile duct formation associated with ALGS causes poor excretion of bile, which leads to cholestasis, elevated liver function tests, and hypercholesterolemia [1]. Jaundice with pruritus and xanthomas can occur secondary to this hyperbilirubinemia and hypercholesterolemia [1–3]. Severity of liver disease varies among patients; cholestasis typically worsens until school age and may resolve in some patients [9]. A small fraction of patients (~15%) will have progressive disease leading to cirrhosis and liver failure that requires transplantation [2–4].

Our patient had paucity of intrahepatic bile ducts on liver biopsy along with chronic cholestasis, congenital heart disease, and characteristic dysmorphic facies, meeting the classic criteria for ALGS. Her cardiac presentation is somewhat consistent with previously reported presentations of congenital heart disease in ALGS, which occurs in about 90% of patients [1–4, 8]. The most frequent cardiovascular lesions include peripheral pulmonary artery stenosis, pulmonary atresia, Tetralogy of Fallot (TOF), patent ductus arteriosus, ASD, and VSD. Some degree of pulmonary outflow tract hypoplasia occurs in the majority of cases [1–4, 8, 10]. Other anomalies include truncus arteriosus, coarctation of the aorta, aortic stenosis, hypoplastic left heart syndrome, and aneurysms of the cerebral circulation [1, 3, 4, 8, 10]. Our patient also presented with dysplastic kidneys which, although not a part of the criteria, is common presentation of ALGS especially with* NOTCH2* mutations [1, 3, 5]. Structural problems (hypoplastic kidneys, cysts, and ureteropelvic obstructions) and functional abnormalities (renal tubular acidosis) are the most common renal anomalies [1, 3]. In addition, our patient showed early signs of failure to thrive, which occurs in high proportion of ALGS patients (>80%), with malabsorption being the main contributing factor to the growth retardation [1, 3]. This patient demonstrated the phenotypic variability of ALGS, which can range from minimal apparent clinical symptoms to severe disease requiring organ transplantation to even death [5].

The varied presentation of ALGS is attributed to defects in the Notch signaling pathway (NSP), which is an intracellular signaling system that regulates cellular determination and differentiation in a variety of tissues [1, 3, 5, 6]. NSP is also associated with cardiovascular development and angiogenesis, with mutations thought to cause cardiac defects and vasculopathies [5, 10]. The majority of ALGS cases (90–97%) are due to mutations or deletions at* JAG1* ligand (ALGS type 1), causing protein termination and haploinsufficiency of the gene [1, 4, 5].* JAG1* is located on the long arm of chromosome 20 20p11.2–20p12 [6]. About 1% of cases are due to mutations in* NOTCH2* gene (1p13, ALGS type 2), which are commonly associated with renal defects and less so with heart, facial, or vertebral anomalies [1, 5]. There also exist a 60–70% de novo mutation rate and a relatively high rate of germline mosaicism with reduced penetrance associated with ALGS [1, 5, 6].

Due to a high suspicion for ALGS with our patient, a Cholestasis NextGen Sequencing panel was sent to PreventionGenetics Laboratory. The test method utilized was a combination of Next Generation Sequencing (NGS) and Sanger sequencing technologies. Regions with insufficient coverage by NGS were covered by Sanger sequencing. All pathogenic and novel variants were confirmed by Sanger sequencing.

Our patient was found to be positive for two novel mutations suspected to be pathogenic for ALGS. Her genetic analysis showed heterozygous expression of* JAG1* gene for a sequence variant designated as c.2729dupA. This is predicted to result in a premature protein termination of p.Cys911Valfs^*∗*^41. This variant has not previously been reported but is likely pathogenic and the primary cause of her disease. She was also found to have heterozygous expression of the* NOTCH2* gene for a sequence variant designated as c.4819C>T. This is predicted to result in the amino acid substitution p.Arg1607Cys. The clinical significance of this variant is not clear but may be pathogenic. Of the more than 400 known* JAG1* and* NOTCH2* mutations, both of these variants have not been previously reported in the literature or the general population (http://exac.broadinstitute.org/). Both parents had no history of cardiac or hepatic problems and were supposed to undergo genetic testing. However, the patient passed away before additional genetic analysis could be pursued.

As for therapy, the severity and presentation of ALGS determine the level of treatment, ranging from supportive care to standard medical management for cardiac and hepatic complications, including transplantation and cardiac surgeries in severe cases [1, 3]. Prognosis of ALGS is also related to severity of organ involvement, with congenital heart disease, progressive liver disease, intracranial bleeding, and infection being the main contributors to increased mortality [1–4]. Complex congenital heart disease, specifically TOF, is the most significant indictor of early mortality, while liver complications are attributed to greater proportion of later deaths [1]. Our patient was not considered a candidate for corrective cardiac surgery given the complexity and extent of her disease. Subsequently, her symptoms were managed through supportive measures during her last few months of life.

## 4. Conclusion

Providers should maintain a high level of suspicion for ALGS when newborns present with early-onset jaundice, especially when other characteristic signs of ALGS are present. With regard to cardiac manifestations, there is significant variation in presentation and thorough evaluation is necessary as some defects can be life-threatening, while others are amenable to surgical correction. For our patient, surgery was unfortunately not a viable option and symptomatic management was the best option to improve her quality of life. In terms of the genetic findings, the* JAG1* c.2729dupA (p.Cys911Valfs^*∗*^41) and* NOTCH2* c.4819C>T (p.Arg1607Cys) variants have not previously been described and, with no obvious family history, it is most likely these were de novo mutations. However, it is also crucial to consider that the highly variable expression and reduced penetrance seen with ALGS could be influencing clinical presentation seen in one or both of our patient's parents. Genetic counseling for all families with suspicion or confirmed ALGS is recommended for family planning and diagnosing carrier individuals.

## Figures and Tables

**Box 1:**
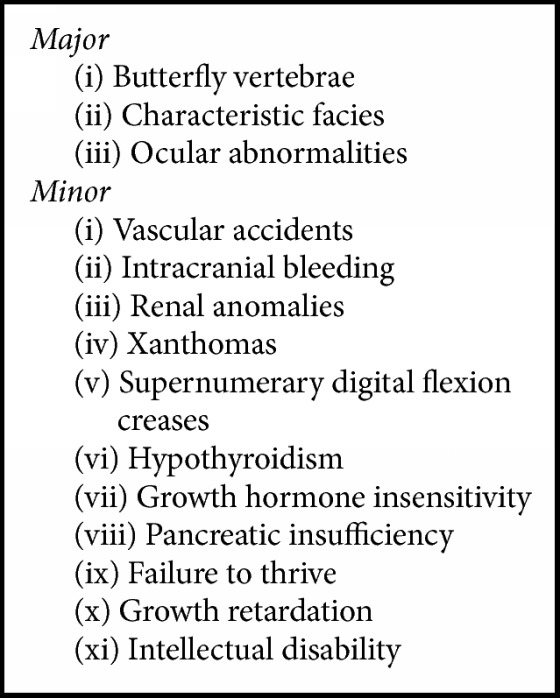
Abnormalities of Alagille Syndrome [1, 3, 4].

**Box 2:**
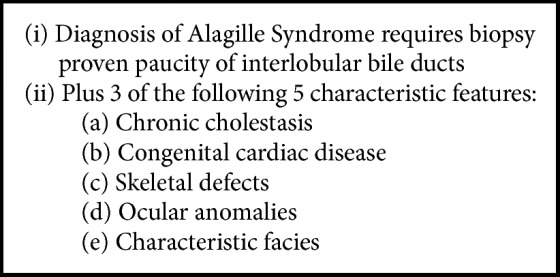
Major diagnostic criteria of Alagille Syndrome [2, 4, 7, 8].

## Abbreviations

ALGS: Alagille Syndrome
ASD: Atrial septal defect
DOL: Day of life
NGS: Next Generation Sequencing
TOF: Tetralogy of Fallot
VSD: Ventricular septal defect.
